# Serological testing of cattle experimentally infected with *Mycoplasma mycoides *subsp. *mycoides *Small Colony using four different tests reveals a variety of seroconversion patterns

**DOI:** 10.1186/1746-6148-7-72

**Published:** 2011-11-18

**Authors:** Evelyn Schubert, Konrad Sachse, Jörg Jores, Martin Heller

**Affiliations:** 1 National Reference Laboratory for CBPP, Friedrich-Loeffler-Institut (Federal Research Institute for Animal Health), Naumburger Str. 96a, 07743 Jena, Germany; 2 Institute of Molecular Pathogenesis, Friedrich-Loeffler-Institut (Federal Research Institute for Animal Health), Naumburger Str. 96a, 07743 Jena, Germany; 3 International Livestock Research Institute (ILRI), Old Naivasha Road, P.O. Box 30709, 00100 Nairobi, Kenya; 4 Institute of Bacterial Infections and Zoonoses, Friedrich-Loeffler-Institut (Federal Research Institute for Animal Health), Naumburger Str. 96a, 07743 Jena, Germany

## Abstract

**Background:**

To study the specific antibody response to infection with *Mycoplasma mycoides *subsp. *mycoides *Small Colony (*Mmm*SC), the agent of Contagious Bovine Pleuropneumonia (CBPP), we examined three panels of sera collected during three experimental infection trials in African cattle. The methods used included an in-house complement fixation test (CFT), a commercially available CFT, a competitive antibody ELISA (cELISA) and the immunoblotting test (IBT). In addition, lung tissue samples were examined by culture.

**Results:**

A total of 89% (51/59) of all experimentally infected animals tested positive on at least one of the serological tests throughout the trial. The specific antibody titres to the *Mmm*SC infection became positive first by CFT (6 to 9 days post infection [dpi]), followed by IBT (9 to 13 dpi) and cELISA (13 to 16 dpi). Individual animals were found to display remarkably distinct seroconversion patterns, which allowed their classification into i) early high responders, ii) late high responders, and iii) low responders. In accordance with other studies, none of the present serological tests was capable of detecting all CBPP infected animals.

**Conclusion:**

Comparison of the assays' performance in terms of sensitivity and specificity raises serious questions as to their reliability for identification of infected individuals in the field. In view of these limitations, a combination of CFT and cELISA can markedly improve CBPP diagnosis at single-animal level.

## Background

Contagious Bovine Pleuropneumonia (CBPP) caused by *Mycoplasma mycoides *subsp. *mycoides *Small Colony (*Mmm*SC), is a highly contagious respiratory disease notifiable to the World Organization for Animal Health (Office International des Epizooties, OIE). While the disease is an immediate threat to livestock producers in the endemic regions of Africa, its implications in terms of epidemiology and animal health affect other geographical areas as well. Characteristic symptoms include anorexia, fever and respiratory signs, such as dyspnoea, polypnoea, cough and nasal discharge. In Africa, the disease has been spreading due to economic, climatic and political factors, and the limitations of currently available diagnostic tests have often been detrimental to efficient control efforts. As for Europe, which has been free of the disease since 1999 [1], the risk of CBPP re-introduction through clinically inconspicuous carrier animals is still existing and deserves permanent attention of traders and importers in the face of intensive international trade in live cattle.

Despite their known limitations, serological methods are still the first choice for herd diagnosis of CBPP, with the complement fixation test (CFT) and a competitive enzyme-linked immunosorbent assay (cELISA) being listed as official methods in the OIE Manual [2].

The CFT, first described in 1953 [3], is widely used in countries struggling with the disease, and a modified "micro method" is common in countries of the European Union [2]. It is assumed that some of the CFT's drawback in terms of specificity can be overcome by using the cELISA [4]. The immunoblotting test (IBT), which has been recommended by the OIE [2] as an alternative in case of ambiguous results from CFT or ELISA, was shown to be highly specific and sensitive [5].

Furthermore, according to OIE recommendations, suspected CBPP cases identified by serology should be confirmed by specific detection of the pathogen. While culture is a cumbersome and time-consuming procedure requiring fresh tissue samples, PCR allows specific identification of the pathogen within hours. High sensitivity is characteristic for optimised PCR assays, with detection limits between 10 [6] and 100 colony-forming units [7]. More recently, several real-time PCR assays for *Mmm*SC were described [8-12]. However, if used for herd diagnosis, PCR-based tests have their limitations due to intermittent shedding of the pathogen, restricted access to mycoplasmas in sequestra and the absence of adequate equipment outside central laboratories.

Apart from the ongoing discussion on the choice of the diagnostic test, the time course and dynamics of the specific antibody response in infected animals is not well documented. In a typical clinical case, the major pathological consequence of *Mmm*SC infection is a massive inflammatory reaction mainly restricted to the lungs of the affected animal [13], which is associated with a rise in specific antibodies. However, it was mentioned that individual animals respond rather differently to challenge infection and vaccination [14-16], thus suggesting different immune response patterns. In addition, the existence of symptomless carriers in the field is known, some of which may be in the sub-acute or chronic phase of infection presenting low titres or none at all [17].

It was the aim of the present study to monitor seroconversion of cattle experimentally challenged with *Mmm*SC. For this purpose, we compared the results from four serological assays and assessed their suitability for testing at single-animal and herd levels.

## Methods

### Bovine sera

#### Panel 1 (End point sera from B237 trial)

Thirty Zebu cattle were experimentally infected with *Mmm*SC B237, a strain isolated from an outbreak in Thika, Kenya in 1997, as described previously [18]. The animals were observed for up to 47 days (see Table 1), and sera were collected during post mortem examination. Re-isolation of the agent was conducted and pathological findings were specified in 15 animals (505, 506, 509, 513, 515, 519, 520, 522, 525, 527, 532, 539, 542, 543, and 544). Three negative cattle sera originated from the animal facility of FLI Jena, Germany. Positive control serum for the in-house CFT (reference serum no. 315) and positive control serum for the IBT were kindly provided by J. Regalla, LNIV Lisbon, Portugal.

**Table 1:** Results of the B237 infection trial (Panel 1): Serological testing, re-isolation of *Mmm*SC and pathological findings from challenged cattle*

**Animal-No**.	in-house CFT		CIRAD CFT		cELISA		IBT	Re-isolation	Days after infection at slaughter	Lung lesions (size)
	Titer	Result	Titer	Result	Inhibition [%]	Result				
505	0	-	0	-	28.7	-	+	yes	44	RC (9 cm)
506	0	-	0	-	34.0	-	-	yes	47	no lesion
509	0	-	10	+	62.3	+	+	yes	29	whole left side with consolidation
513	640	+	160	+	41.6	amb	+	yes	29	whole lung with consolidation
515	5	-	0	-	29.1	-	+	yes	44	no lesion
519	160	+	80	+	70.1	+	+	yes	47	RC and RD (9 cm each)
520	0	-	0	-	20.4	-	amb	no	47	no lesion
522	160	+	40	+	56.7	+	+	yes	47	whole left side with consolidation
525	160	+	40	+	51.2	+	+	yes	29	whole lung
527	320	+	80	+	61.6	+	+	yes	44	RA (5 cm)
532	0	-	0	-	46.7	amb	-	yes	47	fibrous adhesions
539	0	-	0	-	19.5	-	amb.	yes	47	no lesion
542	1280	+	640	+	42.5	amb	+	yes	29	RA and RC (9 cm each)
543	160	+	80	+	54.0	+	+	yes	44	RA and RC (9 cm each)
544	0	-	0	-	32.1	-	+	yes	44	no lesion

* All animals that were examined pathologically have been included (15/30 animals).

RA = right apical lobe, RC = right cadiac, RD = right diaphragmatic, amb = ambiguous (aberration from standard band pattern).

The CFT titre is given as the inverse of the highest dilution yielding 100% inhibition of haemolysis.

#### Panel 2 (Sera from short-term Afadé trial)

The animals were experimentally infected with *Mmm*SC, strain Afadé, a strain isolated from an outbreak in Northern Cameroon in 1968 (kindly provided by Joachim Frey, University of Berne). Briefly, each animal was inoculated intrabronchially with 50 ml of fresh *Mmm*SC broth culture (2.5 × 10^10 ^colony forming units per animal), followed by 20 ml of liquid 1.5% agar solution and 30 ml phosphate-buffered saline. Sera were taken periodically every 3 to 4 days from the day before infection (-1 dpi) until necropsy, i.e. up to 30 dpi, from 20 Boran cattle (BD091-102, BD105-107, BD111, BD115, BD116, BD118, BD119). Positive and negative control sera were the same as in Panel 1. Infection mode, sampling scheme, clinical symptoms and pathological observations have been described elsewhere [18,19].

#### Panel 3 (Sera from long-term Afadé trial)

To obtain samples from the late and chronic stages of the disease, sera were taken periodically on a weekly basis from 7 experimentally infected Boran cattle (BD103, BD104, BD108, BD109, BD112, BD114, and BD117) over a time period of approximately 8 months to obtain samples from the late and chronic stages of the disease. Infection mode and challenge strain were the same as in Panel 2.

### Tissue samples

Lung tissue samples were collected upon necropsy and used for examination by culture and PCR [18,19]. Lungs were inspected for lesions and, where possible, material from these areas was excised for pathogen detection.

The animal experiments mentioned in this paper were conducted in strict accordance with Kenyan legislation on animal experimentation and were approved by the Institutional Animal Care and Use Committee (IACUC reference no. 2008.08). ILRI has been voluntarily complying with the United Kingdom's Animals Act 1986, which contains guidelines and codes of practice for housing and care of animals used in scientific procedures.

### Complement fixation tests

The protocols of the two CFTs used in this study were largely identical, but notably differed in the type of antigen used and the conditions of the antigen-binding step.

a) The in-house CFT was carried out in microplate format as recommended by the OIE for detection of antibodies to *Mmm*SC. Phenol-inactivated whole-cell antigen of the type strain *Mmm*SC PG1, which was previously checkerboard titrated, was used as antigen at a concentration of 2 complement fixing units. Other reagents for the in-house CFT complement, i.e. haemolytic serum, veronal buffer and sheep red blood cells, were obtained from Virion-Serion (Würzburg, Germany). Antigen binding was allowed during overnight incubation at 4°C.

b) The CIRAD CFT (CIRAD, Montpellier, France), was conducted according to the manufacturer's instructions. The kit included all reagents except veronal buffer, sheep red blood cells and negative control serum (purchased from Virion-Serion). The incubation time for antigen binding at 37°C was 30 min.

All sera used were inactivated at 56°C for 30 min and diluted in the range of 1:5 to 1:2560. The highest dilutions of sera producing 100% haemolysis inhibition of sheep red blood cells were taken as end points of dilutions to be examined, and CFT titres were given as reciprocals of these dilutions.

CFT readings were scored according the OIE manual [2], i.e. positive in the case of 100% inhibition of haemolysis at a serum dilution of 1:10 or greater; ambiguous at 25, 50 or 75% inhibition at 1:10 serum dilution, and negative with absent haemolysis or haemolysis at 1:5 serum dilution.

### Enzyme immunoassay

The CBPP serum competitive ELISA (IDEXX, Institute Pourquier, Montpellier, France) was used for screening the sera. The test is based on the paper by Le Goff and Thiaucourt [20] and uses a monoclonal anti-*Mmm*SC antibody, as well as microplates coated with *Mmm*SC lysate. The ELISA was performed according to the instructions of the manufacturer. Optical densities (OD) were measured at 450 nm using the photometer Spectra Fluor (Tecan, Crailsheim, Germany). The percentage inhibition value (INH%) for each serum sample was calculated using the formula:

$$\text{INH\% }=\left(\text{O}{\text{D}}_{\text{mab}}-\text{O}{\text{D}}_{\text{sample}}\right)∕\left(\text{O}{\text{D}}_{\text{mab}}-\text{O}{\text{D}}_{\text{conjugate}}\right)\times \text{1}00\%,$$

$$\text{O}{\text{D}}_{\text{mab}}=\text{Control only with monoclonal antibody and without serum }\left(0\%\text{ inhibition}\right)$$

$$\text{O}{\text{D}}_{\text{sample}}=\text{OD of the serum sample}$$

$$\text{O}{\text{D}}_{\text{conjugate}}=\text{Control without monoclonal antibody and serum }\left(\text{1}00\%\text{ inhibition}\right).$$

The cut-off for positive samples was at INH% of 50%. Sera with an inhibition value between 40% and 50% were considered doubtful. All sera were examined in duplicate.

### Immunoblotting test

The IBT was performed according to Regalla et al. [5] and the OIE manual [2] with minor modifications. SDS-PAGE (7.5% polyacrylamide) separated proteins of reference strain *Mmm*SC PG1 and strain Afadé were transferred onto nitrocellulose membrane (Whatman, Dassel, Germany). The BenchMark Protein Ladder 10-220 kDa (Invitrogen, Karlsruhe, Germany) was used as molecular weight marker. To control the efficiency of protein transfer, a reversible protein staining step using Ponceau S (Sigma-Aldrich, Taufkirchen, Germany) was included. Excised membrane strips were incubated with 1:100 serum dilutions or control serum, respectively. Reactive bands were visualised using alkaline phosphatase-conjugated recombinant protein A/G (Pierce, Bonn, Germany) and substrate BCIP/NTB (5-bromo-4-chloro-3-indolyl phosphate combined with nitrotetrazolium blue chloride, Sigma-Aldrich). Positive sera were expected to show a specific pattern that included reactive bands at 110, 98, 95, 60/62, and 48 kDa. IBT patterns of sera were scored ambiguous in case that either the specific band of 98 kDa was missing or other specific bands were weak.

### Bacteriological evaluation and identification

Isolation and cultivation of *Mycoplasma *strains from tissue samples were performed using standard methodology [21]. Lung tissue samples collected during post mortem from margins of sequestra or regions of necrosis were inoculated into modified Hayflick medium [21] and incubated at 37°C for 3 to 4 days. Identification of *Mmm*SC from these cultures was done using PCR (see next paragraph).

### PCR

Mycoplasma cultures and tissue samples of Panel 2 and 3 animals were examined using a *Mmm*SC-specific PCR assay described previously [6,22].

### Statistical evaluation

The kappa agreement test was conducted [23] to compare the concordance between individual tests. For each animal, the mean of all the measurements was calculated for each individual assay and an animal was classified positive based on the set criterion. The proportion of positive animals was computed as a percentage of the test positives against the total number tested.

## Results

### Examination of end point sera from the B237 trial (Panel 1)

Of the 30 animals challenged, 25 (83.3%) displayed a specific antibody response detected by at least one of the serological tests, whereas all sera from non-infected animals remained negative. A positive test result of sera to all the four tests was observed in 12 (40%) cases. Re-isolation of the challenge strain was attempted in 15 cases, of which 14 were successful [18]. The findings obtained from these animals are summarised in Table 1. Notably, the infective strain was also re-isolated from four animals without lesions. The lesion-free subgroup tested negative both in cELISA and CFTs (animals 506, 515, 520, 539, 544), IBT was either positive (animals 515 and 544), ambiguous (animals 520 and 539; Table 1), or negative (animal 506). Regarding all infected animals, the number of positive results in IBT was higher than in the other methods.

### Examination of sera from the short-term Afadé trial (Panel 2)

**Figure 1:**
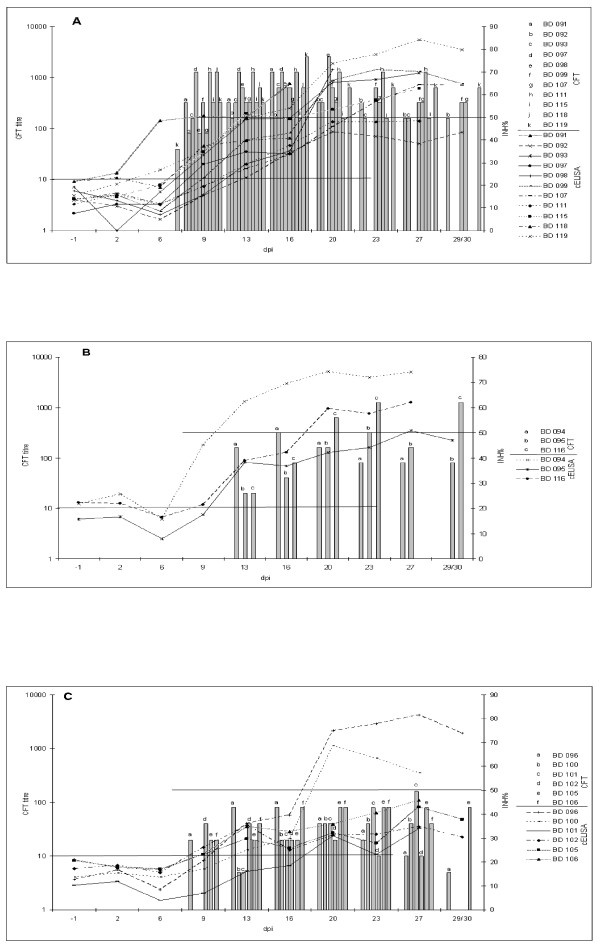
**Comparison of CIRAD CFT and cELISA-based curves showing the antibody response of animals from Panel 2**. Animals from the Short-term Afadé trial were characterised by CFT (bars) and cELISA (curves). Horizontal lines represent the cut-off values of CFT (dilution 1:10, 100% inhibition of haemolysis) and cELISA (50% inhibition), respectively. The assignment of letters and symbols to the respective animals is given in the right-hand column. A: Early high responders (according to CFT), B: Late high responders, C: Low responders. Missing bars at later time points indicate that the respective animal died or had to be removed prematurely.

Serological testing using both CFTs confirmed successful infection of all animals (see Additional File 1: Results of in-house CFT vs. CIRAD CFT from sera of Panel 2). The time course of specific antibody production in all 20 animals is shown in Figure 1. The humoral response patterns as detected by CFT can be classified into three categories, **i) **early high responders (BD091, BD092, BD093, BD097, BD098, BD099, BD107, BD111, BD115, BD118, BD119), **ii) **late high responders (BD094, BD095, BD116), and **iii) **low responders (BD096, BD100, BD101, BD102, BD105, BD106). CFT titres of early high responders emerged at 6 dpi in the case of animal BD119 and at 9 dpi in the other animals (Figure 1A). For late high responders, a pronounced rise around 13 dpi was characteristic (Figure 1B).

**Figure 2:**
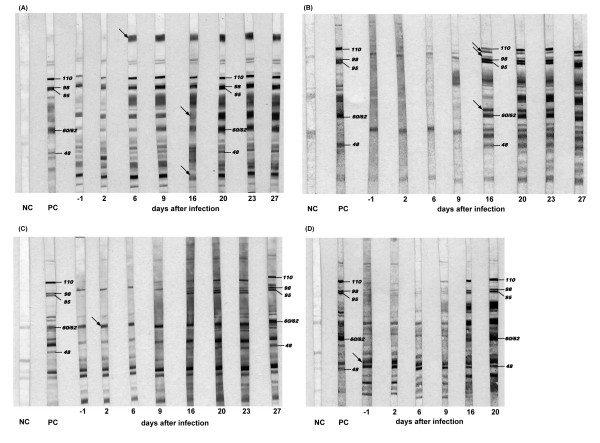
**Immunoblotting analysis of sera from selected animals of Panel 2 (Short-term Afadé trial)**. Nitrocellulose strips carrying SDS-PAGE separated whole-cell antigen of *Mmm*SC strain Afadé were incubated with a 1:100 dilution of the serum, and reactive bands were visualised using AP-conjugated anti-bovine IgG and BCIP/NTB. A: animal BD093, B: animal BD099, C: animal BD101, D: animal BD097. Black arrows indicate additional bands close to *Mmm*SC-specific bands that are not observed with all sera (B, C, and D) and a prominent additional band, which occurred only on positive strips of animal BD093 (A). Molecular weights (in kDa) of specific bands that have been recommended for identification of the infection are indicated. NC = negative control serum, PC = positive control serum.

Figure 1 also reveals that the course of the humoral responses of Panel 2 cattle were not completely concurrent in CFT and cELISA. The increase of the cELISA titres was seen after day 6 post infection (see Additional File 2: Examination of Panel 2 sera using cELISA), with the exception of animal BD091 (beginning at 6 dpi, Figure 1A; see also below). In the subgroup of low responders, the rise in cELISA titres was generally weak, with animals BD101, BD102, BD105, and BD106 remaining below the cut-off level throughout the trial (as did animals BD092, BD097, BD111, and BD118 of the early high responder group). Nevertheless, the cELISA-negative animals mentioned showed positive results in both CFTs (see Additional File 1: Results of in-house CFT vs. CIRAD CFT from sera of Panel 2). In addition, the cELISA-negative animals BD097 and BD101 exhibited characteristic bands in the IBT. Figure 2 shows immunoblot patterns of four selected animals of Panel 2. While the IBT results were largely comparable to CFT findings, this test also detected emerging specific antibodies to *Mmm*SC at earlier time points than cELISA (Table 2).

**Table 2:** Comparison of the specific antibody response of selected* cattle from the short-term Afadé trial (Panel 2) using four serological tests

Animal	Time of serum collecion (dpi)	In-house CFT	CIRAD CFT	cELISA	IBT
	-1	-	-	-	-
	2	-	-	nd	nd
	6	-	-	-	-
	9	+	+	-	amb
BD 093	13	+	+	amb	+
	16	+	+	amb	+
	20	+	+	+	+
	23	+	+	+	+
	27	+	+	+	+

	-1	-	-	-	-
	2	-	-	-	-
BD 097	6	-	-	-	-
	9	+	+	-	amb
	13	+	+	-	+
	16	+	+	-	+

	-1	-	-	-	-
	2	-	-	-	-
	6	-	-	-	-
	9	+	+	-	-
BD 099	13	+	+	amb	nd
	16	+	+	amb	+
	20	+	+	+	+
	23	+	+	+	+
	27	+	+	+	+

	-1	-	-		-
	2	-	-	-	-
	6	-	-	-	-
	9	-	-	-	-
BD 101	13	-	-	-	nd
	16	+	+	-	amb
	20	+	+	-	+
	23	+	+	-	+
	27	+	+	-	+

	-1	-	-	-	nd
	2	-	-	-	nd
	6	-	-	-	nd
	9	-	+	-	nd
BD 105	13	-	+	-	nd
	16	-	+	-	nd
	20	-	+	-	nd
	23	-	+	-	nd
	27	+	+	amb	nd

	-1	-	-	-	nd
	2	-	-	-	nd
BD 118	6	-	-	-	nd
	9	+	+	-	nd
	13	+	+	-	nd
	16	+	+	amb	nd

* animals surviving at least until 16 dpi, amb = ambiguous, nd = not done

Seropositivitiy of Panel 2 sera in cELISA and CFT did not always correlate with clinical symptoms and patho-morphological signs. Conversely, however, negative or weakly positive readings in cELISA and CFT correlated with the absence of clinical signs or mild symptoms. Patho-morphological lesions typical for *Mmm*SC infections were observed in all animals of the short-term Afadé trial except animal BD102. Four animals died or had to be sacrificed prematurely, i.e. at 16 dpi (BD091, BD097, BD118) or at 20 dpi (BD098). This subgroup belonged to early high responders showing severe clinical symptoms (high temperature, cough, dyspnoea), as well as a variety of patho-morphological lesions in lung and lymph node tissues.

### Examination of sera from the long-term Afadé trial (Panel 3)

**Figure 3:**
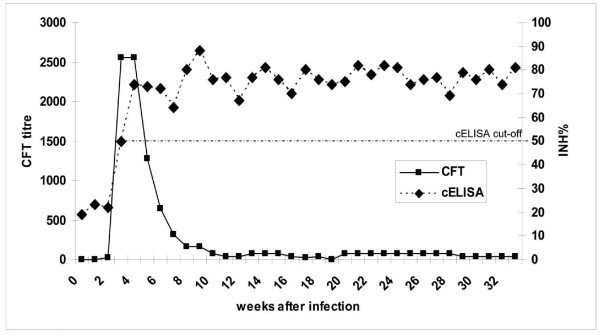
**Comparison of CIRAD CFT and cELISA based curves showing the specific antibody response of animal BD114**. CFT titres of animal BD114 (Panel 3, long-term Afadé trial) are given as the inverse value of the highest dilution yielding 100% inhibition of haemolysis. Please note, that the CFTs cut-off is at 10 (dilution of the serum 1:10).

Sera from all 7 animals showed positive reactions with all four methods used. In a typical time course (Figure 3), detectable CFT titres emerged in week 2 p.i. and decreased steadily from weeks 5-8. At the end of this infection trial, i.e. 34 weeks p.i., CFT titres ranged between 1:10 to 1:20 for the in-house CFT (see Additional File 3: Examination of Panel 3 sera using in-house CFT), and between 1:5 and 1:160 for the CIRAD CFT (see Additional File 4: Examination of Panel 3 sera using CIRAD CFT). Since sera were collected only on a weekly basis, classification into responder groups as in Panel 2 animals was not feasible. Notably, animal BD117 differed from the other animals by responding one week later in both CFTs.

Test results of the cELISA showed a rise in titres from week 3 to 4 p.i., with levels either remaining positive until to the end of the trial (Figure 3) or slowly decreasing to reach the cut-off value of 50% inhibition (see Additional File 5: Examination of Panel 3 sera using cELISA).

**Figure 4:**
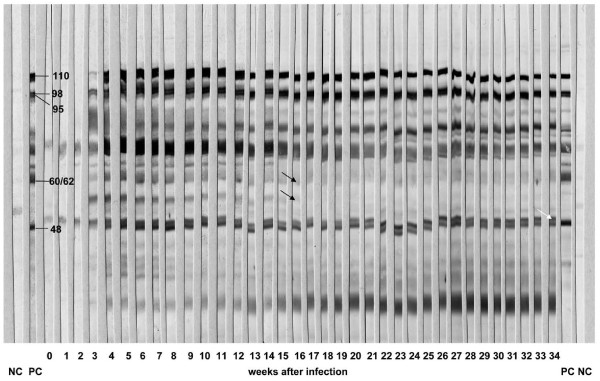
**Immunoblotting analysis of animal BD103 (Panel 3, long-term Afadé trial)**. Western blotted nitrocellulose strips of *Mmm*SC strain Afadé were incubated with 1:100 dilutions of sera collected weekly from challenged animal BD103 (Panel 3) and processed as described in Materials and Methods. The white arrow indicates an additional band close to the specific 48-kDa band, and black arrows indicate bands fading after week 15 p.i. Molecular weights (in kDa) of specific bands that have been recommended for identification of the infection are indicated. NC = negative control serum, PC = positive control serum.

The time course of specific antibody response as monitored by IBT is shown in Figure 4. Animal BD103 was somewhat peculiar as the specific antigenic band of 60-62 kDa faded after week 10 p.i. The same band disappeared in the reaction of animal BD117 after week 10, whereas animal BD104 maintained the entire specific banding pattern until the end at week 34 (data not shown).

### General comparison between the serological methods

All test results of the four assays from sera of Panels 1 and 2 have been compared and summarised in Table 3. The compilation shows that general agreement among the results of all tests used was poor. As confirmed by kappa agreement testing, the results of in-house and CIRAD CFTs were always close to each other, when taking into account the inherent systematic error of two dilution titres (see Table 1, see Additional File 1: Results of in-house CFT vs. CIRAD CFT from sera of Panel 2). The number of positive findings by CFTs was generally higher than those from cELISA, which implies a delay in the detection of the onset of antibody production by the latter (see Table 2, Figure 1). The sensitivity of the IBT proved intermediate between cELISA (less sensitive) and CFT (more sensitive), when consecutively collected sera were examined (Panel 2).

**Table 3:** Examination of Panel 1 and 2 sera: Comparison of all serological tests*

Assays compared	Number (percentage) of animals reacting similarly in the tests compared		
	30 sera of Panel 1	Selection of 30 sera of Panel 2 (including IBT)	All 178 sera of Panel 2 (without IBT)
cELISA and in-house CFT	21 (70%), ĸ = 0,46^c^	18 (60%), ĸ = 0,33^d^	114 (70%), ĸ = 0,43^c^
cELISA and CIRAD CFT	23 (77%), ĸ = 0,58^c^	18 (60%), ĸ = 0,33^d^	108 (62%), ĸ = 0,38^d^
In-house CFT and CIRAD CFT	27 (90%), ĸ = 0,79^a^	30 (100%), ĸ = 1,00^a^	161 (90%), ĸ = 0,80^a^
In-house CFT and IBT	21 (70%), ĸ = 0,39^d^	26 (87%), ĸ = 0,33^d^	
CIRAD CFT and IBT	21 (70%), ĸ = 0,39^d^	26 (87%), ĸ = 0,33^d^	
cELISA and IBT	17 (57%), ĸ = 0,23^d^	19 (63%), ĸ = 0,38^d^	
In-house CFT, CIRAD CFT and cELISA	21(70%)	18 (60%)	102 (59%)
In-house CFT, CIRAD CFT, cELISA and IBT	15 (45%)	18 (60%)	

* Kappa agreement testing [23] was additionally conducted. According to standard criteria, the concordance between two tests compared is classified as ^a ^very good (1-0.76), ^b ^good (0.75-0.61), ^c ^acceptable (0.6-0.4), ^d ^poor (< 0.4).

## Discussion

The remarkable degree of variation in the humoral immune response displayed by the animals of the present study indicates a high complexity of host-pathogen interactions during *Mmm*SC infection, which can lead to acute, sub-acute to chronic or symptomless courses of disease [17]. On the one hand, the immune status of the individual animal seems to play a role in the time course and level of specific antibody production. Naïve animals are assumed to react in a different fashion than re-infected cattle exhibiting an anamnestic response, and symptomless carriers can exhibit low antibody levels in the absence of intense host pathogen interactions. Inter-animal differences in the cellular immune response [18,19], which have not been addressed in the present study, may also add to the overall diversity observed.

On the other hand, the pathogen has been shown to possess a genetically determined machinery for surface antigen variation [24,25], which enables it to evade the host immune response by selecting modified phenotypes that cannot be challenged by cognate antibodies. Depending on the efficiency of the individual host defence, the progress of *Mmm*SC infection can be expected to vary from animal to animal.

The authors wish to emphasise that the present comparative analysis of diagnostic tests is referring to the individual animal level, which is a limitation because the data cannot be simply extrapolated to herd level. While currently available serological tests are generally suitable for herd diagnosis, the present findings highlight serious limitations of these tests at the individual animal level, which have to be taken into account when field studies are conducted.

CFT titres do not represent the whole spectrum of specific antibodies present in the infected animal, nor are they long lasting. The half-life of CFT antibody titres was estimated to be approximately 30 days [20,26]. Notably, our own data indeed show a steep decline in these titres, beginning between weeks 3 and 6 p.i. (Figure 3, see Additional File 3: Examination of Panel 3 sera using in-house CFT and Additional File 4: Examination of Panel 3 sera using CIRAD CFT). Such a drop in CFT titres was also observed in the contact challenge study of Niang and co-workers [17], albeit significantly delayed (from week 16 to 36) as the precise time point of each individual infection remained elusive in that infection model. Taken together, these observations imply that field studies based solely on CFT are prone to miss individual animals at the later stages of infection.

While the differences between the two CFTs used were marginal, the relative diagnostic sen-sitivity of both tests compared to culture was 50.0% (in-house CFT) and 57.1% (CIRAD CFT) with Panel 1 samples, which is in line with data of other authors [27]. The consistently observed divergence between the results of CFT and cELISA (Table 2, Figure 3) is probably a consequence of the different immunoglobulin classes covered by each method. Thus, IgG class antibodies have a greater affinity in the cELISA, while IgG2 subclass antibodies are unable to fixate complement used in the CFT. Moreover, IgM class antibodies, which are characteristic for early infection, are easier to detect by CFT [28,29]. This can explain the earlier detection of antibodies by CFT as observed in the present study and elsewhere [20,30]. Furthermore, the present finding that IgG antibody levels from cELISA remained at a high level for a prolonged period is in agreement with the study by Niang and co-workers [17], where the kinetics of different antibody isotypes was investigated.

It is important to note that the present cELISA was given a relatively high cut-off in order to maximise specificity, which in turn diminishes the test's sensitivity [31]. In fact, the present evidence suggests that there is some room for lowering the cut-off without loss of specificity. However, this has to be confirmed by further studies involving more field sera from cattle herds having CBPP and/or other mycoplasma infections. We hypothesise that another way to improve the test's performance includes the use of specific peptides [32] or recombinant *Mmm*SC proteins [16,33] instead of whole-cell antigen.

The IBT has been described as being more sensitive and specific than CFT [13], which has been confirmed by the present data (Table 1). Thus, the IBT showed specific reaction patterns for sera tested negative in CFT and cELISA (animals 515, 520, 539, 544, 546). The test's high specificity is based on the reaction to five different antigens, i.e. 110, 98, 95, 60/62, and 48 kDa proteins, which must be recognised by their specific antibodies in order to identify a positive serum [2]. However, we observed some problems with the test's reproducibility and potential for standardisation, as immunoblot reaction patterns are complex and individual bands may be difficult to identify when non-specific bands from cross-reactions with other bacteria are interfering. Cross-reactions with closely related *Mycoplasma *(*M*.) species of the "mycoides cluster" seem to be less important here, because they are rarely encountered in cattle, but other mycoplasmas, such as *M. bovis *and *M. bovigenitalium*, may play a role [13,31].

Culture of *Mmm*SC from affected lung tissue was included to underpin our serological findings. While seropositive animals always showed clinical symptoms of CBPP that were confirmed by pathology, the presence of lung lesions was no guarantee for successful re-isolation of the challenge strain (Panel 2, data not shown). The findings of the present study also suggest that re-isolation was seriously hampered at the late and chronic stages of infection, i.e. isolation of *Mmm*SC did not succeed from tissue samples of Panel 3 animals after 35 weeks p.i. The absence of a strict correlation was particularly evident with animal 506 (Panel 1, Table 1), where all serological tests were negative (and pathological signs were missing) despite successful re-isolation of the pathogen.

## Conclusions

The present study has revealed three distinct seroconversion patterns among *Mmm*SC-infected animals, i) early high responders, ii) late high responders, and iii) low responders. This variability raises questions as to the choice and suitability of current serological tests for single-animal diagnosis. While valid at the herd level, individual test results can be misleading and negative serological findings should be interpreted with particular caution.

Two factors account for the lack of sensitivity of serological tests at the single-animal level, i) titres of specific antibodies at an early stage of infection and in chronic carriers can be very low, and ii) high variability in antigen expression by *Mmm*SC *in vivo*, where not all relevant proteins are expressed at a given point in time [24,34]. We suggest that diagnostic testing should comprise both CFT and cELISA, particularly in countries declared free of CBPP.

Unlike CFT and ELISA, the IBT requires experienced and well-trained laboratory personnel and is not suitable for use in routine laboratories. To improve reproducibility, we recommend **i**) the use of 7.5% acrylamide gels instead of gradient gels as prescribed in the OIE manual [2], and **ii**) the use of the same antigen in all laboratories, i.e. *Mmm*SC strain Afadé. We propose that the methodology in [2] be accordingly revised and supplemented with more detailed instructions to address the above mentioned problems (see Additional File 6: Proposal for modification of the current OIE protocol for IBT), so that IBT can be used as an additional test in the case of ambiguous CFT and/or cELISA results.

## Abbreviations

BCIP/NTB: 5-bromo-4-chloro-3-indolyl phosphate combined with nitrotetrazolium blue chloride; CFT: complement fixation test; cELISA: competitive enzyme linked immunosorbent assay, CBPP: Contagious Bovine Pleuropneumonia; dpi: days post infection; IBT: immunoblotting test; kDa: kilodalton; MmmSC: Mycoplasma mycoides subsp. mycoides Small Colony; OD: optical densities; PCR: polymerase chain reaction; INH%: percentage inhibition value; p.i.: post infection; OIE: Office International des Epizooties (World Organization for Animal Health)

## Competing interests

The authors declare that they have no competing interests.

## Authors' contributions

ES provided and supported laboratory work, evaluated and interpreted the data and wrote the manuscript. KS has been involved in supporting laboratory work and in discussion of results including revising the manuscript. JJ designed and coordinated the animal experiments using the Afadé strain and provided serum and tissue samples, MH coordinated the investigation, evaluated and interpreted all data of CFT, immunoblotting test, PCR, and culture investigations. All authors revised the manuscript and approved the final version.

## Supplementary Material

Additional file 1 **Results of in-house CFT vs. CIRAD CFT from sera of Panel 2**. Humoral immune response of animals from Panel 2 (Short-term Afadé trial) as characterised by in-house CFT and CIRAD CFT. End-point titres of both CFTs are shown at different time points of infection. Click here for file

Additional file 2 **Examination of Panel 2 sera using cELISA**. Humoral immune response of animals from Panel 2 (Short-term Afadé trial) characterised by cELISA. The percentage inhibition value (INH%) for each serum sample was calculated using the formula: INH% = (OD_mab _- OD_sample_)/(OD_mab _- OD_conjugate_) × 100%, OD_mab _= Control only with monoclonal antibody and without serum (0% inhibition), OD_sample _= OD of the serum sample, OD_conjugate _= Control without monoclonal antibody and serum (100% inhibition). The cut-off for positive samples was set at INH% of 50%. Sera with an inhibition value between 40% and 50% were considered doubtful. All sera were examined in duplicate. ND = not done. Click here for file

Additional file 3 **Examination of Panel 3 sera using in-house CFT**. Examination of the 7 sera from the long-term Afadé trial (Panel 3) using in-house CFT. End-point titres of the CFT were shown until 34 weeks p.i. Click here for file

Additional file 4 **Examination of Panel 3 sera using CIRAD CFT**. Examination of the 7 sera from the long-term Afadé trial (Panel 3) using CIRAD CFT. End-point titres of the CFT were shown until 34 weeks p.i. Click here for file

Additional file 5 **Examination of Panel 3 sera using cELISA**. Examination of the 7 sera from the long-term Afadé trial (Panel 3) using cELISA. Data of the cELISA were given in percentage inhibition and shown until 34 weeks p.i. Click here for file

Additional file 6 **Proposal for modification of the current OIE protocol for IBT**. To improve the reproducibility of IBT results, the authors of the present paper recommend two modifications to the protocol of the OIE Manual. Click here for file
