# Supplementary material for: Serological testing of cattle experimentally infected with Mycoplasma mycoides subsp. mycoides Small Colony using four different tests reveals a variety of seroconversion patterns
Source: BMC Vet Res. 2011 Nov 18;7:72. doi: 10.1186/1746-6148-7-72 (PMC3377920; doi:10.1186/1746-6148-7-72)
Supplement: Additional file 1 — Results of in-house CFT vs. CIRAD CFT from sera of Panel 2. Humoral immune response of animals from Panel 2 (Short-term Afadé trial) as characterised by in-house CFT and CIRAD CFT. End-point titres of both CFTs are shown at different time points of infection. [file 1746-6148-7-72-S1.DOC]

Results of in-house CFT vs. CIRAD CFT from sera of Panel 2 (end-point titres)

| dpi  Animal | -1 | | 2 | | 6 | | 9 | | 13 | | 16 | | 20 | | 23 | | 27 | | 29 | | 30 | |
| --- | --- | --- | --- | --- | --- | --- | --- | --- | --- | --- | --- | --- | --- | --- | --- | --- | --- | --- | --- | --- | --- | --- |
|  | In-house | CIRAD | In-house | CIRAD | In-house | CIRAD | In-house | CIRAD | In-house | CIRAD | In-house | CIRAD | In-House | CIRAD | In-house | CIRAD | In-house | CIRAD | In-house | CIRAD | In-house | CIRAD |
| BD 091 | - | - | - | - | - | - | 640 | 320 | 640 | 320 | 640 | 1280 |  |  |  |  |  |  |  |  |  |  |
| BD 092 | - | - | - | - | 20 | - | 160 | 80 | 160 | 160 | 160 | 160 | 320 | 320 | 320 | 320 | 640 | 160 | 640 | 160 |  |  |
| BD 093 | - | - | - | - | - | - | 80 | 160 | 80 | 320 | 640 | 640 | 320 | 320 | 80 | 160 | 80 | 160 |  |  |  |  |
| BD 094 | - | - | - | - | - | - | 10 | - | 80 | 160 | 160 | 320 | 320 | 160 | 320 | 80 | 160 | 80 |  |  |  |  |
| BD 095 | - | - | - | - | - | - | - | - | - | 20 | - | 40 | 160 | 160 | 160 | 320 | 640 | 160 |  |  | 320 | 80 |
| BD 096 | - | - | - | - | - | - | 80 | 20 | 40 | 80 | 40 | 80 | 20 | 40 | 20 | 20 | 20 | 10 |  |  | 20 | 5 |
| BD 097 | - | - | - | - | - | - | 320 | 1280 | 640 | 1280 | 640 | 1280 |  |  |  |  |  |  |  |  |  |  |
| BD 098 | - | - | - | - | - | - | 40 | 80 | 320 | 640 | 320 | 640 | 640 | 2560 |  |  |  |  |  |  |  |  |
| BD 099 | - | - | - | - | - | - | 320 | 320 | 1280 | 320 | 1280 | 640 | 320 | 640 | 640 | 640 | 320 | 320 |  |  | 320 | 320 |
| BD 100 | - | - | - | - | - | - | - | - | - | 5 | 20 | 20 | 80 | 40 | 40 | 40 | 40 | 40 |  |  |  |  |
| BD 101 | - | - | - | - | - | - | - | - | - | 5 | 20 | 20 | 20 | 40 | 40 | 80 | 80 | 160 |  |  |  |  |
| BD 102 | - | - | - | - | - | - | 10 | 40 | 10 | 40 | 10 | 20 | 10 | 20 | 10 | 10 | 10 | 10 |  |  | 10 | 0 |
| BD 105 | - | - | - | - | - | - | - | 20 | - | 20 | - | 20 | - | 80 | - | 80 | 80 | 80 | 80 | 80 |  |  |
| BD 106 | - | - | - | - | - | - | - | 20 | - | 40 | 20 | 80 | 40 | 80 | 40 | 80 | 40 | 40 |  |  |  |  |
| BD 107 | - | - | - | - | - | - | - | 80 | - | 320 | 80 | 320 | 80 | 320 | 80 | 320 | 80 | 320 |  |  | 80 | 320 |
| BD 111 | - | - | - | - | - | - | 640 | 1280 | 640 | 1280 | 640 | 1280 | 640 | 1280 | 640 | 1280 | 640 | 1280 |  |  |  |  |
| BD 115 | - | - | - | - | 10 | - | 80 | 320 | 160 | 320 | 160 | 160 | 320 | 160 | 320 | 160 | 320 | 160 |  |  |  |  |
| BD 116 | - | - | - | - | 5 | - | 20 | - | 80 | 20 | 160 | 80 | 320 | 640 | 640 | 1280 |  |  | 1280 | 1280 |  |  |
| BD 118 | - | - | - | - | 5 | - | 640 | 1280 | 640 | 640 | 320 | 640 |  |  |  |  |  |  |  |  |  |  |
| BD 119 | - | - | - | - | 10 | - | 160 | 320 | 160 | 320 | 320 | 2560 | 320 | 640 | 320 | 640 | 640 | 640 | 1280 | 640 |  |  |
