# Supplementary material for: Serological testing of cattle experimentally infected with Mycoplasma mycoides subsp. mycoides Small Colony using four different tests reveals a variety of seroconversion patterns
Source: BMC Vet Res. 2011 Nov 18;7:72. doi: 10.1186/1746-6148-7-72 (PMC3377920; doi:10.1186/1746-6148-7-72)
Supplement: Additional file 2 — Examination of Panel 2 sera using cELISA. Humoral immune response of animals from Panel 2 (Short-term Afadé trial) characterised by cELISA. The percentage inhibition value (INH%) for each serum sample was calculated using the formula: INH% = (ODmab - ODsample)/(ODmab - ODconjugate) × 100%, ODmab = Control only with monoclonal antibody and without serum (0% inhibition), ODsample = OD of the serum sample, ODconjugate = Control without monoclonal antibody and serum (100% inhibition). The cut-off for positive samples was set at INH% of 50%. Sera with an inhibition value between 40% and 50% were considered doubtful. All sera were examined in duplicate. ND = not done. [file 1746-6148-7-72-S2.DOC]

Examination of Panel 2 sera using cELISA (in INH%)

| dpi  Animal | -1 | 2 | 6 | 9 | 13 | 16 | 20 | 23 | 27 | 29 | 30 |
| --- | --- | --- | --- | --- | --- | --- | --- | --- | --- | --- | --- |
| BD 091 | 21,7 | 25,6 | 48,6 | 50,7 | 49,7 | 64,8 |  |  |  |  |  |
| BD 092 | 13,4 | 10,6 | 4,9 | 15,5 | 23,3 | 34,4 | 43,6 | 41,5 | 38,4 | 43,3 |  |
| BD 093 | 19,3 | ND | 17,1 | 33,3 | 49,2 | 49,6 | 65,4 | 66,7 | 69,6 |  |  |
| BD 094 | 21,8 | 25,7 | 15,9 | 45,2 | 62,4 | 69,6 | 74,2 | 72,0 | 74,1 |  |  |
| BD 095 | 15,8 | 16,6 | 7,9 | 17,5 | 38,4 | 36,9 | 42,2 | 44,2 | 50,9 |  | 47,1 |
| BD 096 | 12,8 | 16,6 | 8,4 | 20,5 | 36,3 | 39,6 | 75,0 | 77,8 | 81,6 |  | 74,0 |
| BD 097 | 7,5 | 11,6 | 11,7 | 29,3 | 34,7 | 33,8 |  |  |  |  |  |
| BD 098 | 17,5 | 13,2 | 7,0 | 15,9 | 29,6 | 35,2 | 71,1 |  |  |  |  |
| BD 099 | 13,6 | 15,6 | 8,4 | 23,2 | 40,1 | 43,1 | 66,3 | 70,9 | 70,1 |  | 64,6 |
| BD 100 | 13,7 | 15,4 | 13,6 | 17,1 | 25,1 | 29,7 | 68,6 | 63,4 | 57,2 |  |  |
| BD 101 | 10,2 | 11,8 | 3,9 | 7,0 | 16,1 | 18,5 | 31,1 | 23,2 | 34,0 |  |  |
| BD 102 | 17,3 | 18,8 | 15,5 | 26,2 | 35,4 | 25,1 | 31,3 | 31,7 | 34,8 |  | 30,5 |
| BD 105 | 20,6 | 17,9 | 17,0 | 23,2 | 29,8 | 25,8 | 32,3 | 27,9 | 43,1 | 37,8 |  |
| BD 106 | 21,0 | 17,6 | 16,7 | 23,2 | 34,9 | 32,8 | 36,0 | 40,7 | 45,8 |  |  |
| BD 107 | 13,7 | 16,5 | 11,9 | 19,5 | 27,2 | 33,6 | 45,9 | 56,9 | 64,3 |  | 64,1 |
| BD 111 | 11,6 | 15,9 | 11,5 | 19,6 | 29,2 | 37,6 | 47,9 | 47,9 | 48,5 |  |  |
| BD 115 | 14,1 | 14,8 | 20,1 | 34,6 | 51,6 | 49,2 | 53,5 | 57,7 | 62,6 |  |  |
| BD 116 | 22,4 | 22,0 | 16,5 | 21,5 | 39,0 | 42,1 | 59,6 | 57,6 | ND | 62,1 |  |
| BD 118 | 21,7 | 23,4 | 19,2 | 37,3 | 39,8 | 40,7 |  |  |  |  |  |
| BD 119 | 15,5 | 20,5 | 26,7 | 35,2 | 49,3 | 54,1 | 73,8 | 77,8 | 84,1 | 79,7 |  |
