# Supplementary figures and images for: Serological testing of cattle experimentally infected with Mycoplasma mycoides subsp. mycoides Small Colony using four different tests reveals a variety of seroconversion patterns
Source: BMC Vet Res. 2011 Nov 18;7:72. doi: 10.1186/1746-6148-7-72 (PMC3377920; doi:10.1186/1746-6148-7-72)

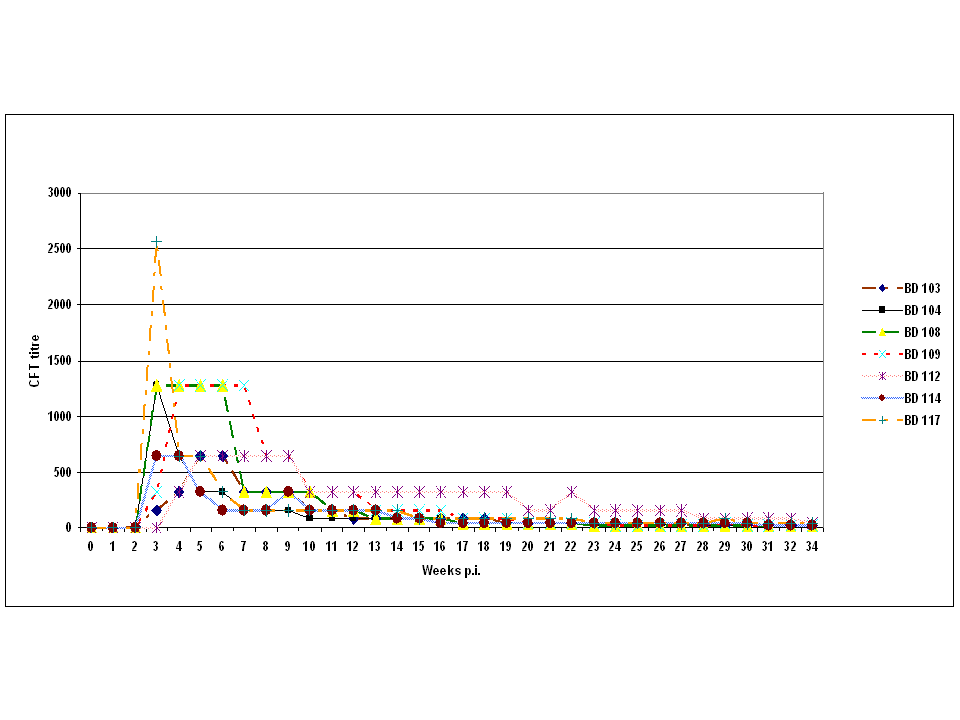

Supplement: Additional file 3 — Examination of Panel 3 sera using in-house CFT. Examination of the 7 sera from the long-term Afadé trial (Panel 3) using in-house CFT. End-point titres of the CFT were shown until 34 weeks p.i. [file 1746-6148-7-72-S3.TIFF]

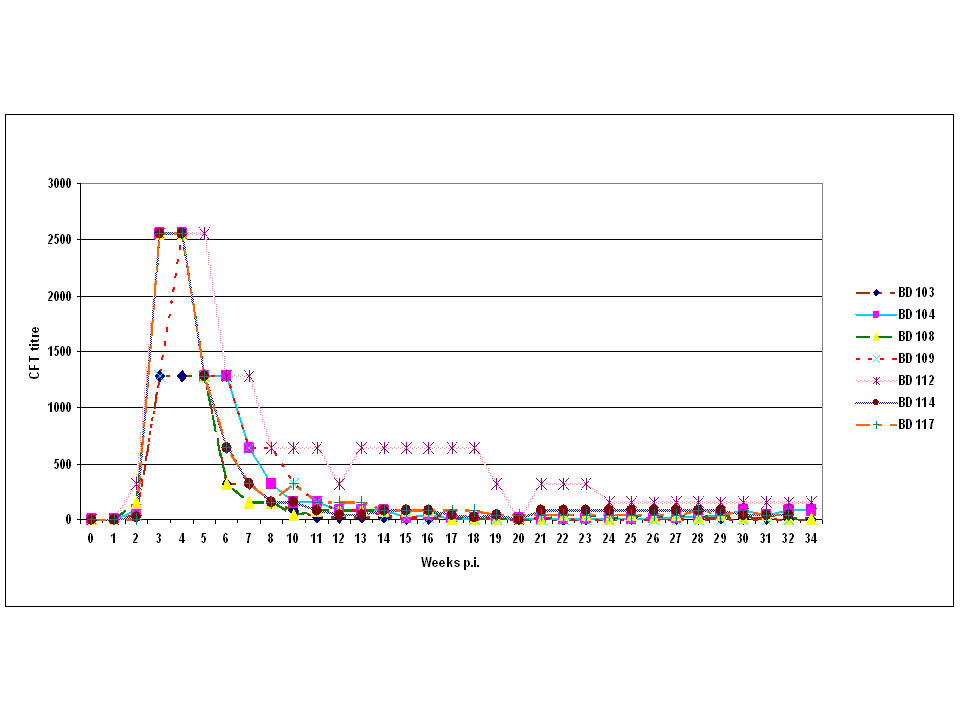

Supplement: Additional file 4 — Examination of Panel 3 sera using CIRAD CFT. Examination of the 7 sera from the long-term Afadé trial (Panel 3) using CIRAD CFT. End-point titres of the CFT were shown until 34 weeks p.i. [file 1746-6148-7-72-S4.TIFF]

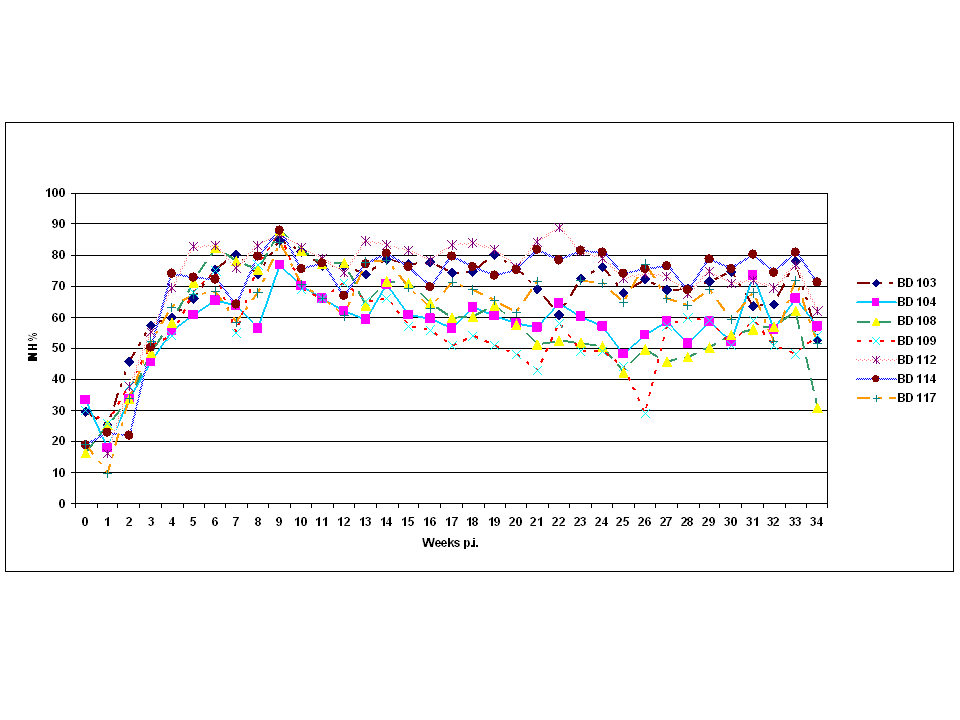

Supplement: Additional file 5 — Examination of Panel 3 sera using cELISA. Examination of the 7 sera from the long-term Afadé trial (Panel 3) using cELISA. Data of the cELISA were given in percentage inhibition and shown until 34 weeks p.i. [file 1746-6148-7-72-S5.TIFF]
