# Supplementary material for: Serological testing of cattle experimentally infected with Mycoplasma mycoides subsp. mycoides Small Colony using four different tests reveals a variety of seroconversion patterns
Source: BMC Vet Res. 2011 Nov 18;7:72. doi: 10.1186/1746-6148-7-72 (PMC3377920; doi:10.1186/1746-6148-7-72)
Supplement: Additional file 6 — Proposal for modification of the current OIE protocol for IBT. To improve the reproducibility of IBT results, the authors of the present paper recommend two modifications to the protocol of the OIE Manual. [file 1746-6148-7-72-S6.DOC]

**Proposal for modification of the current OIE protocol for IBT**

To improve the reproducibility of IBT results, the authors of the present paper recommend the following modifications to the protocol of the OIE Manual [1].

**1. Use 7.5 % acrylamide gels for SDS-PAGE (no gradient).**

The present version of the OIE manual [1] recommends the use of a 5 to 15% SDS gradient gel for PAGE separation of *Mmm*SC whole-cell proteins. Since distinction between the main antigenic bands at 98 and 95 kDa appeared to be a major problem, we conducted the IBT at an acryl amide concentration of 7.5% and observed good resolution of all specific antigenic bands.

**2. All laboratories conducting IBT should use the same antigen. We suggest *Mmm*SC strain Afadé.**

The instructions for the IBT in the OIE Manual do not specify the strain to be used as antigen in SDS-PAGE. In view of the known differences between African and European strains of *Mmm*SC [2] it seems important for the reproducibility of the results that the same *Mmm*SC strain and the same protein preparation procedure are used. Indeed [2] and [3] already mentioned that not all strains showed the 98-kDa band. Thus, the Italian strain *Mmm*SC L2 lacked this band when used with homologous and heterologous sera, and the same band was also missing when other *Mmm*SC strains were used as antigen to test sera from animals infected with *Mmm*SC L2.

**References**

1. **Manual of diagnostic tests and vaccines for terrestrial animals.** 6th Edition, 2008 [http://www.oie.int/fileadmin/Home/eng/Health_standards/tahm/2.04.09_CBPP.pdf]

2. Gonçalves R, Regalla J, Nicolet J, Bashiruddin J, De Santis P, Penha Gonçalves, A: **A 98 kDa antigenic band as a possible epidemiological marker differentiating European isolates of *Mycoplasma mycoides* subsp. *mycoides* SC.** In: *COST 826-Mycoplasma of ruminants: Pathology, Diagnostics, Epidemiology and Molecular Genetics*. Edited by Frey J, Sarris, K. Luxembourg: European Commission; 1996:49-51.

3. Abdo EM, Nicolet J, Miserez R, Goncalves R, Regalla J, Griot C, Bensaide A, Krampe M, Frey J: **Humoral and bronchial immune responses in cattle experimentally infected with Mycoplasma mycoides subsp. mycoides small colony type.** *Vet Microbiol* 1998, **59:**109-122.
